# Advanced materials for enamel remineralization

**DOI:** 10.3389/fbioe.2022.985881

**Published:** 2022-09-13

**Authors:** Jiarong Xu, Hui Shi, Jun Luo, Haiyan Yao, Pei Wang, Zhihua Li, Junchao Wei

**Affiliations:** ^1^ School of Stomatology, Nanchang University, Nanchang, Jiangxi, China; ^2^ Jiangxi Province Clinical Research Center for Oral Diseases, Nanchang, China; ^3^ Jiangxi Province Key Laboratory of Oral Biomedicine, Nanchang, Jiangxi, China

**Keywords:** advanced materials, enamel caries, demineralization, remineralization, hydroxyapatite

## Abstract

Dental caries, a chronic and irreversible disease caused by caries-causing bacteria, has been listed as one of the three major human diseases to be prevented and treated. Therefore, it is critical to effectively stop the development of enamel caries. Remineralization treatment can control the progression of caries by inhibiting and reversing enamel demineralization at an early stage. In this process, functional materials guide the deposition of minerals on the damaged enamel, and the structure and hardness of the enamel are then restored. These remineralization materials have great potential for clinical application. In this review, advanced materials for enamel remineralization were briefly summarized, furthermore, an outlook on the perspective of remineralization materials were addressed.

## 1 Introduction

The enamel, consisting of 96–97 wt% inorganic hydroxyapatite (HA, Ca_10_(PO_4_)_6_(OH)_2_), 3wt% water and 1wt% organic material, is the hardest tissue in the human body (Bowen et al., 2018; Harper et al., 2021). However, enamel is susceptible to acid, causing enamel demineralization and even developing cavities (Pitts et al., 2017). Currently, hundreds of millions of people in the world is under the enamel damage (Peres et al., 2019). It is difficult to repair enamel on its own due to the lack of sufficient calcium and phosphate ions in saliva (Lawn et al., 2010; Lacruz et al., 2017). Therefore, artificial materials such as resin, metal or bioglass are commonly used for clinical repair of cavities (Dorri et al., 2017). In terms of composition, mechanical properties, and appearance, these composites differ significantly from enamel. By comparison, enamel remineralization can be an effective clinical method for restoring the natural properties and structure of enamel while avoiding the problems associated with filling materials. Remineralization requires replacing minerals lost during the early stages of demineralization to restore enamel hardness or structure.

**FIGURE 1:**
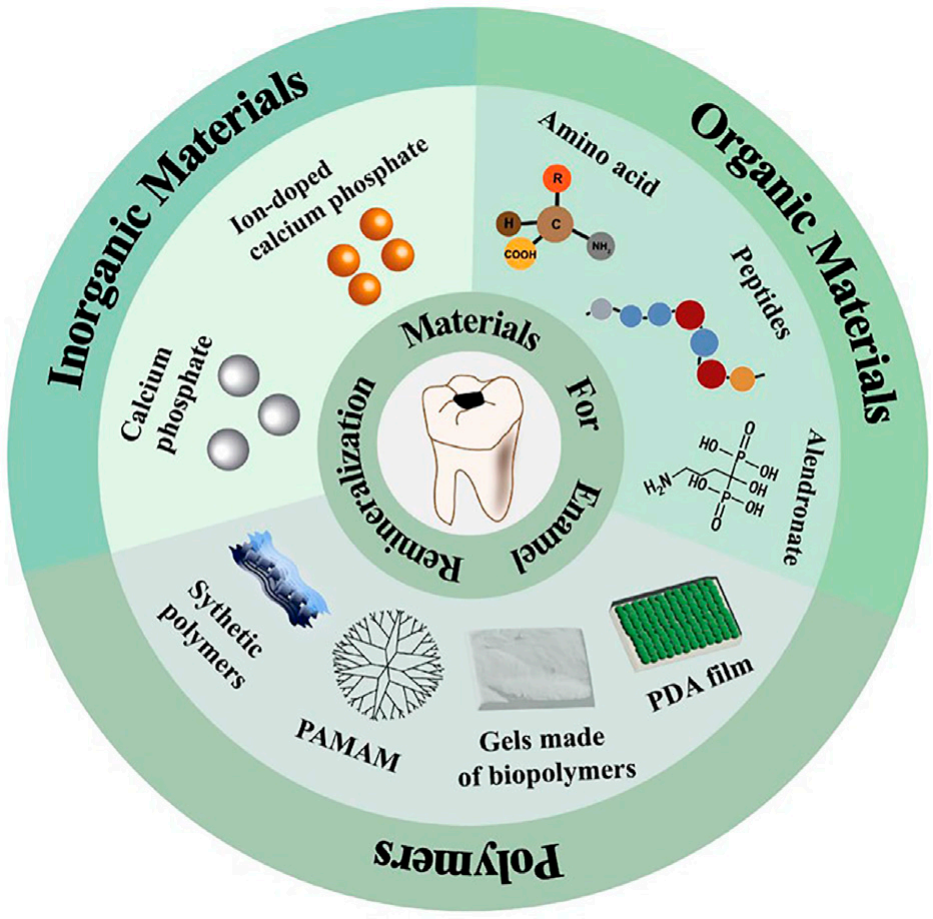
Schematic diagram of advanced materials promoting remineralization and repairing enamel.

Remineralized materials are essential to enamel repair. Functional materials can promote and arrange the deposition of calcium and phosphate ions or alter the solubility of the HA. They can be divided into inorganic materials, organic materials, and polymeric materials (Figure 1). These functional materials are designed to rebuild remineralized tissue on damaged enamel surfaces, thereby preventing disease progression while also improving aesthetics and mechanical strength. Therefore, materials for enamel remineralization have a bright future in clinic. Although several reviews of remineralized materials have been published (Cochrane et al., 2010; Ding et al., 2017; Pandya and Diekwisch, 2019), enamel remineralized materials have been innovated and developed. As a result, it is critical to review the relevant research progress in time for the construction and upgrading of the enamel remineralization system. In this review, the characteristics and working mechanism of remineralized materials are briefly summarized. The specific functions of various functional materials will be clarified by category, with reference opinions provided for future material design and synthesis.

## 2 Functional inorganic materials

Functional inorganic materials can induce the formation of apatite layers or release ions, which can promote the remineralization of enamel. When the remineralized layer forms, calcium phosphates (CaPs) provide exogenous ions to compensate minerals lost by enamel, while fluoride and magnesium ions can exchange with calcium ions in HA, changing the solubility and mechanical properties of ion-doped HA. Therefore, the ability of inorganic materials to release ions and the change in enamel properties caused by their participation in HA are the primary focal point of researches.

### 2.1 Calcium phosphates

CaPs can provide ions to reconstruct damaged enamel. Remineralization solutions containing calcium and phosphorus ions are usually used in remineralization experiments, which must be replaced or replenished on a regular basis. Some stable CaPs materials can provide ions required for an extended time. Amorphous calcium phosphate (ACP), tricalcium phosphate (TCP), and nano-hydroxyapatite (nHA) are common CaPs materials used for remineralization. The type and size of the CaPs crystals can influence the ion supply capacity and the depth of ion entry into the lesion. Therefore, the mineralization effects of these materials are differrent.

ACP, the precursor phase of biogenic HA of bone and tooth, is the basic mineralization unit in the biological mineralization process (Gelli et al., 2019). Aqueous ACP solutions contain abundant Ca^2+^ and PO_4_^3−^ ions, which form highly hydrated clusters. The structure and composition of the crystalline phase change after further aggregation of clusters until the thermodynamically stable crystalline HA (alkaline conditions) or carbon brushes (acidic conditions) formed (He et al., 2020). Usually, such reaction time is fast in the absence of external interference. Only ACP solutions failed to restore enamel (Shao et al., 2019). Therefore, enamel remineralization requires ensuring the stability of ACP in solution and prolonging its phase transition time. Acidic groups, such as carboxyl and phosphoric groups, can bind calcium ions in solution, preventing Ca^2+^ and PO_4_^3−^ from aggregating. Organic compounds with carboxyl or phosphate groups are the most common ACP stabilizers. It is a good method to use amino acids such as aspartate (Asp), glutamate (Glu), citrate (Delgado-López et al., 2014; Iafisco et al., 2015), and the phosphate stabilizer triethylamine (Shao et al., 2019) to maintain the size of ACP particles, ensuring ion supply in the subsequent mineralization process. In addition, the casein phosphopeptide (CPP) that containing four to seven phosphate groups can attach to ACP nanoclusters, forming CPP-ACP. CPP-ACP complexes have been used as common additives for caries prevention. Furthermore, CPP-ACP in combination with fluoride show advantages in remineralization of existing lesions (Bijle et al., 2018; Tao et al., 2018). However, CPP-containing products should be used with caution in individuals with lactose intolerance issues.

TCP can be divided into *α*-TCP and *β*-TCP according to the crystal form. *β*-TCP is often used in dental materials because of its great biodegradability and biocompatibility. When exposed to acid, *β*-TCP degrades to release ions for enamel restoration. After surface functionalization by carboxylic acid and surfactants, functionalized TCP (fTCP) can prevent fluoride from binding with calcium ions on the enamel surface prematurely to build a low-dose fluoride release system (Karlinsey and Pfarrer, 2012; Shen et al., 2018; Viana et al., 2020). After combining with fumaric acid, fTCP can show significantly higher calcium bioavailability than *β*-TCP and better remineralization of subsurface enamel damage (Karlinsey et al., 2010).

nHA is a bioactive and biocompatible material with a small particle size of 10–20 nm in diameter and 60–80 nm in length (Huang et al., 2011). The nanometer size enables nHA to penetrate deeper lesion layers through large lesion pores and repair enamel damage (Juntavee et al., 2018; Bossu et al., 2019; Memarpour et al., 2019). However, high-concentrating nHA tend to self-aggregate into large-sized nHA, which can affect the amount and depth of nHA entering the lesion (Huang et al., 2009). As a carrier, the gel effectively extends the contact time between the active ingredient and the enamel, allowing nHA to fill the small holes and depressions. Both silica-based glycerol hydrogel (Khonina et al., 2020) and carbomer-based gel (Sari et al., 2021) containing nHA can repair damaged enamel.

### 2.2 Fluorinated compounds

Fluorinated compounds have been commonly utilized since the previous century to reverse or prevent enamel defects from spreading. Consequently, the global incidence rate of dental caries has decreased dramatically (Jokstad, 2016; Clark et al., 2020). Fluoride reduces demineralization by altering enamel solubility (Lynch et al., 2004). Fluoride and calcium ions are more strongly bound than hydroxyl groups. Therefore fluoride can replace hydroxyl to form fluorapatite (FAP), which has high acid resistance and poor solubility (Clark et al., 2020). Fluorides, on the other hand, can promote remineralization by encouraging Ca^2+^ in saliva to attach to the tooth surface. In addition, fluoride can reduce the adhesion and growth of germs by blocking the activities of numerous enzymes.

Fluoride is primarily ingested through drinking water (75%). Fluoridation of home water is a typical measure to prevent dental caries in many countries, and it can successfully reduce the incidence of dental caries. Fluoride can also be found in a variety of oral care treatments and dental materials, including sodium fluoride (NaF), stannous fluoride, silver diamine fluoride, acidulated phosphate fluoride, ammonium fluoride, and others (Barrera-Ortega et al., 2020). Fluoride sustained-release ability could be altered by combining fluoride ions with different cations and complexing it with different organic molecules. In toothpaste and rinses, polyvalent fluorides with tin and titanium as cations exhibit excellent corrosion resistance (Zanatta et al., 2020). This is due to the fact that they can not only produce CaF_2_ on the enamel surface, but can also generate metallic precipitates on the enamel surface, which contributes to the reduction of calcium ion loss when subjected to external erosion.

**FIGURE 2:**
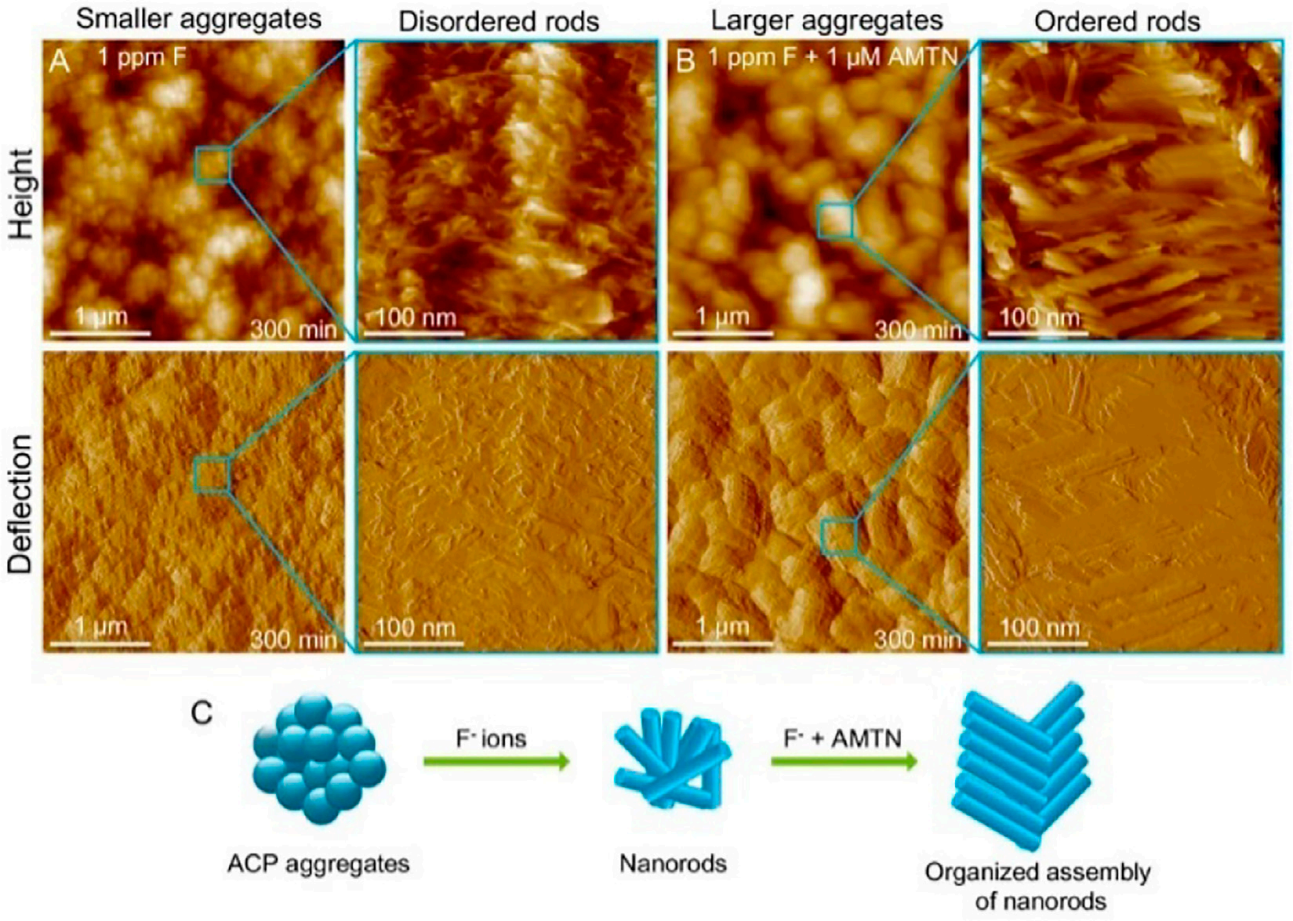
AFM height and deflection images of NPF solution in the presence of **(A)** 1 ppm F and **(B)** 1 ppm F and 1 *μ*M AMTN for 300 min. **(C)** Schematic representation of nanorod tissue assembly in the presence of F ions and AMTN. Reprinted with permission from [ (Yu et al., 2019) ]. Copyright ^©^ 2019 American Chemical Society.

However, fluorides have caused certain issues when they are used. Fluoride tends to develop a disordered layered structure of remineralization layer, which is considerably different from natural enamel. The mechanical characteristics of the remineralized layer can be weakened by these disordered formations. Organic compounds like amelogenin can help minimize the occurrence of disordered structures in the reaction system (Yu et al., 2019) (Figure 2). Moreover, excessive fluoride use can result in hazardous effects like dental fluorosis and skeletal fluorosis (Philip, 2019). It also has the possibility to make cariogenic bacteria resistant, diminishing the effectiveness of follow-up prophylaxis (Liao et al., 2017). Fluorinated hydroxyapatite is rapidly formed in the superficial enamel layer of very concentrated F^−^ solutions, preventing Ca^2+^ and PO_4_^3−^ from penetrating deeper into the lesion. As a result, subsurface enamel lesions can fail to mineralize adequately. Therefore, fluoride slow-release systems made of copolymer acrylic reservoirs and glass ionomer cement can be great alternatives for promoting enamel mineralization by extending the trailing effect (Chong et al., 2018).

Fluoride compounds, as traditional enamel remineralization materials, have a relatively well-studied mineralization mechanism, which facilitates the development of novel fluoride-mediated remineralization systems. However, the functions of fluoride compounds are still need to be improved, and thus, blends or composites of fluoride compounds, which can combine multifunction together to achieve satisfactory clinical results, are greatly needed in the future.

### 2.3 Magnesium related materials

**FIGURE 3:**
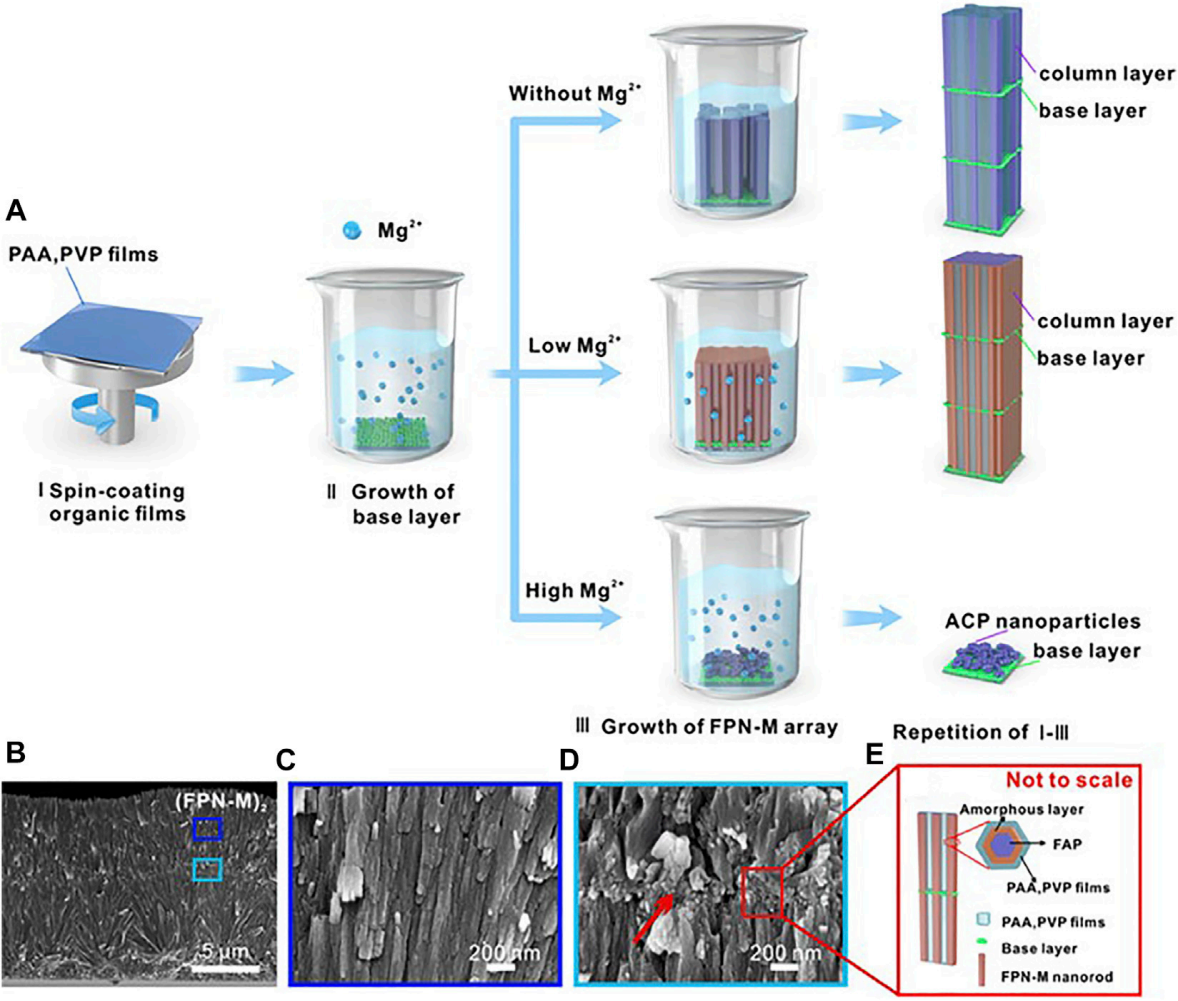
**(A)** The schematic diagram of the synthetic multilayer FAP/polymer nanocomposite controlled by Mg^2+^. **(B)**—**(C)** Cross-sectional SEM images of (FPN-M)_2_ with corresponding details. **(D)** Granular interlaminar base layer (indicated by red arrows). **(E)** Interlayer structure and amorphous-reinforced architecture schematic diagram. Reprinted with permission from [ (Li Y. et al., 2021) ]. Copyright^©^ 2021 American Chemical Society.

Magnesium presents in the hard tissues of the body. In enamel, the content of Mg^2+^ is ranging from 0.2 to 0.5 wt%. Mg^2+^ is present near the grain boundaries as an intergranular phase of Mg substituted amorphous calcium phosphate (Mg-ACP) (La Fontaine et al., 2016). Such amorphous phases have been proved to make a significant impact on the mechanical characteristics and wear resistance of enamel (Gordon et al., 2015). Mg^2+^ slows crystal growth by competing with calcium ions at the growth point during mineralization, affecting the production of apatite (Ren et al., 2010; Abdallah et al., 2016). As a result, Mg^2+^ can act as a competitive inhibitor to guide narrower crystal columns, which promotes a highly ordered arrangement and increases mineralized tissue hardness. As the concentration of Mg^2+^ on the enamel surface increases, the nano-hardness of the enamel rises dramatically (Kis et al., 2021). Layer by layer mineralization process is used to create multilayer arrays of enamel-like FAP/polymer nanocomposites controlled by Mg^2+^ (FPN-M) at room temperature (Li Y. et al., 2021) (Figure 3). In the presence of Mg^2+^, the single nanorods are refined in size and a highly compact array is formed, eventually, (FPN-M)_n_ exhibits excellent mechanical strength and transparency. The present researches have demonstrated Mg^2+^ have great importance during the process of enamel remineralization, therefore, more and more attention should be paid to Magnesium related materials. Besides, further understanding of the relationship between Mg^2+^ and biomineralization can help develop strategies to improve the mechanical properties of mineralized tissues and improve the functions of repaired tooth enamel.

## 3 Functionalized organic materials

Inorganic matter production and growth require a relatively constant environment, which organic materials can offer. Organic molecules are rich in acidic functional groups such as carboxyl, phosphate, and sulfonic acid. These functional groups can induce inorganic compound nucleation, inhibit overgrowth, or interact with hydroxyapatite on the surface of the enamel to increase adsorption capacity. Understanding the specific role of these organic compounds can help to clarify the mechanism of enamel mineralization and provide ideas for future remineralized material design.

### 3.1 Amino acids

Amino acid molecules contain different amounts of amino and carboxyl groups. Depending on the isoelectric point, amino acids can be classified as acidic, neutral, and basic amino acids. Among them, acidic amino acids are negatively charged in weakly acidic solutions, which can influence the nucleation, crystallization, growth, and crystal transformation of HA. Glu and Asp can operate as soft templates, connecting two calcium ions diagonally to generate ordered HA crystals that parallel to the enamel column while stabilizing calcium ions in the solution. The crystals on the enamel surface can grow more ordered with the amino acid concentration rises. Asp and Glu is used to deposit the CaCO_3_ layer as a sacrificial template on the enamel’s surface (Wu et al., 2015). The acidic amino acids then absorbed phosphate and carbonate ions, depositing HA into the CaCO_3_ layer to form the rod crystal. Glycine (Gly), a highly hydrophilic amino acid, can also be used as a biological additive to create enamel-like structures (Tao et al., 2007). According to the molecular dynamics experiment, Gly exhibits the same adsorption abilities and coverage in all directions of the crystal surface, which maintains HA’s *c*-axis propensity (Pan et al., 2007). In the carboxymethyl chitosan-stabilized ACP remineralization system, a rod-shaped crystal layer is successfully produced in artificial caries when Gly is introduced to the system, whereas the system without Gly fail (Wang et al., 2017). Arginine (Arg), a basic amino acid, positively affects pH homeostasis, bacterial ecology, and pathogenicity. Arg is metabolized in oral biofilms to produce ammonia mainly through the internal arginine deiminase system (ADS) of bacteria (*Streptococcus sanguis* and *Streptococcus*). Ammonia produced under this pathway has a significant pH-raising effect, while inhibiting tooth demineralization by neutralizing acids in the peripheral environment (Bijle et al., 2021b). It also facilitates the formation of arginine-friendly microorganisms while disrupting the internal homeostasis of caries-causing bacteria (Nascimento et al., 2019). The combination of Arg and fluoride can create a pH-responsive fluoride pool that inhibit acid production and has potential synergistic effects in maintaining a healthy oral microbial balance (Agnello et al., 2017; Bijle et al., 2021a). The pool can also significantly improve the fluoride uptake and surface hardness of damaged enamel compared to fluoride alone (Zheng et al., 2015; Bijle et al., 2020).

### 3.2 Enamel matrix proteins and proteases

**FIGURE 4:**
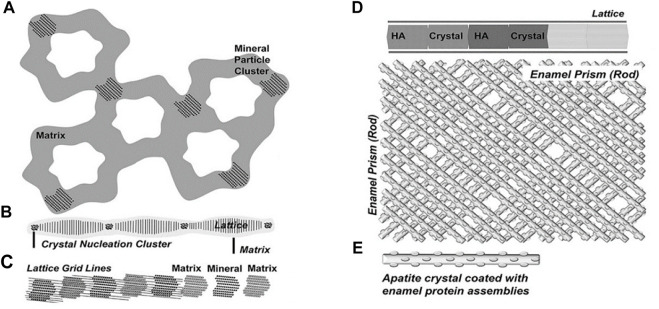
Five successive stages of enamel crystal precipitation and extension. **(A)** Stage of calcium phosphate particles precipitating and adhering to the organized enamel protein matrix, **(B)** Formation of initial crystal needle through single nucleation sites, **(C)** Extension of enamel crystals by lattice-guided alignment of individual apatite crystals, **(D)** Stage of crystal further extension and growing, **(E)** Enamel prisms (rods) are formed by the cross arrangement of single enamel crystals. Reprinted with permission from [ (Jokisaari et al., 2019) ]. Copyright ^©^ 2019 American Chemical Society.

Enamel matrix proteins (EMPs) and proteases control the formation of enamel (Jia et al., 2020; Shin et al., 2020). EMPs govern the parallelism between the glazing columns and organize them in a dense and slender hexagonal prism structure at the micro-level by regulating the creation and structure of HA crystals (Bartlett, 2013; Uskokovic, 2015; Bai et al., 2020). These highly co-oriented glaze columns give enamel its remarkable shear strength and make it resistant to everyday abrasion (Yeom et al., 2017). Over 90% of the EMPs consists of amelogenin. Amelogenin can be enzymatically processed into different peptides. These peptides undergo a change in spatial conformation, manifested by *α*-helix unraveling and *β*-sheet and *β*-turns formation, at which point amyloid-like aggregation occur in the proteins (Carneiro et al., 2016; Bai et al., 2020). Then, they self-assemble into oligomers and nanospheres (Fang et al., 2011; Engelberth et al., 2018; Bai et al., 2020). These oligomeric nanospheres further form nanochains that concentrate Ca^2+^ and PO_4_^3−^ in the peripheral matrix, generating mineralized precursors during enamel development, which then serve as templates to guide the crystal phase transition, eventually generating HA (Gil-Bona and Bidlack, 2020) (Figure 4). The enamel columns then elongate in one direction to form a hexagonal prismatic structure (Jokisaari et al., 2019).

Enzymes are critical requirements for enamel biomineralization. Enzymes activate the biological function of amelogenin and degrade organic matter in the matrix until a sufficiently hard tissue formed (Prajapati et al., 2016). Matrix metalloproteinase 20 (MMP-20) cleaves amelogenin, and the product peptide controls the lengthening and growth of crystal nucleus and induces HA mineralization (Fukae et al., 1998; Nagano et al., 2009; Gil-Bona and Bidlack, 2020). Addition of MMP-20 to full-length porcine amelogenin can promote neatly aligned bundles of enamel-like HA, whereas in the absence of MMP-20, only ACP particles seen (Kwak et al., 2016). Another important enzyme is Kallikrein-related peptidase 4 (KLK4). During enamel maturation, KLK4 degrades the organic matrix in the mineral (Smith et al., 2017; Sari et al., 2021). The width and thickness of the microcrystals can increase when proteins are removed from mature enamel. If the enzyme is deficient, the enamel will undergo hypoplasia (Simmer et al., 2009; Smith et al., 2017).

**FIGURE 5:**
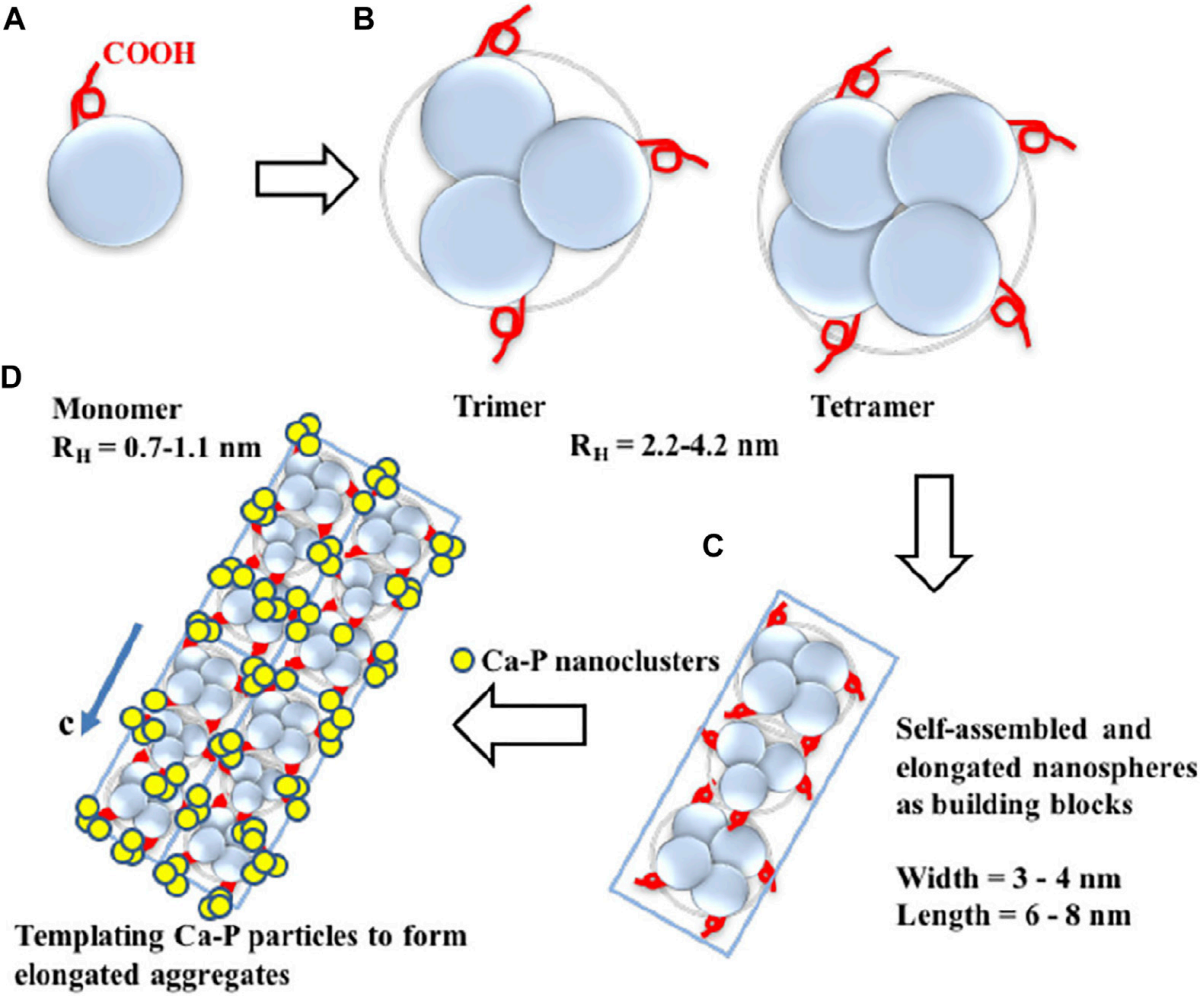
Diagram of amelogenin C-terminal peptides self-assembly to guide the extension of OCP crystallization. **(A)** The monomeric structure of amelogenin’s C-terminal peptide. The red curve represents the -COOH terminus. **(B)** Hydrophobic interactions lead to the formation of oligomeric amelogenin’s C-terminal peptides. **(C)** Nanorod structures as building blocks are formed by the association of oligomers. **(D)** Elongated organic-inorganic complex aggregates formed by the building blocks nanorods and CaP nanoclusters. Reprinted with permission from [ (Wu et al., 2017) ]. Copyright ^©^ 2017 American Chemical Society.

In-depth studies of the enzymatic cleavage products revealed three major functional domains of the amelogenin (Mukherjee et al., 2019; Dissanayake et al., 2020). N-terminal: a hydrophobic tyrosine-rich N-terminal region, known as tyrosine-rich amelogenin peptide (TRAP), is critical in the directed assembly of amelogenin (Buchko et al., 2018). The central region: the central hydrophobic proline-rich region is mainly composed of X-Y-proline (X and Y are usually glutamine) repeat motifs, which is rich in *β*-sheets and *β*-turns. The C-terminal: a highly hydrophilic domain contains a large number of acidic amino acid residues. These residues could combine with Ca^2+^ to provide nucleation sites and bind to the (100) face of octacalcium phosphate (OCP), the intermediate sub-stable phase in early enamel, thereby govern the direction in which the enamel column extends (Wu et al., 2017) (Figure 5). Leucine-rich amelogenin peptide (LRAP), which contains two self-assembled domains of full-length amelogenin, is the most common alternative splicing product of amelogenin (Xia et al., 2016; Green et al., 2019). It is discovered that depending on the phosphorylated version of the peptide on serine 16, LRAP can perform distinct activities. Phosphorylated LRAP (+P) inhibits calcium phosphate crystallization and stabilizes ACP, whereas LRAP (–P) directs the production of aligned enamel crystals (Yamazaki et al., 2017; Le Norcy et al., 2018). *In vitro*, LARP and PP_i_ are used to remineralize the eroded enamel, and acicular HA crystals are successfully regenerated on the surface (Kwak et al., 2017). Inspired by these structural domains, amelogenin analogs are designed and synthesized to induce *in vitro* bionic remineralization. After grafting different fragments, these synthetic functional peptides can be easier to obtain and show certain functional enhancements, such as adsorption ability.

Non-amelogenins work in early enamel formation, including enamelin and tuftelin. Enamelin acts as a transport and nucleation protein that affects amelogenin to regulate early enamel development (Bartlett et al., 2006; Lacruz et al., 2017; Yan et al., 2017). Tuftelin is an acidic protein produced by ameloblasts during the early stages of enamel formation. It is concentrated near the dentin-enamel intersection, in which enamel mineralization begins. The tuftelin-derived peptide (TDP) is created based on the structure of tuftelin. The group repaired by TDP demonstrate comparable enamel hardness and lesion depth healing results after pH cycling to NaF groups (Ding et al., 2020).

### 3.3 Functional peptides

Functional peptides inspired by bioproteins can in some ways replicate the unique functions of these bioproteins, as well as easier access. Assembly of these peptides with different functions can produce multifunctional peptides, such as peptides with high enamel binding and remineralization capacity or peptides with antibacterial and remineralization activities (Table 1).

**TABLE 1:** Synthetic peptides for biomineralization.

Peptide	Sequence	Remineralization effect	References
Chimeric peptide	SVSVGMKPSPRP-GGGGS- LEAWPATDKTKREEVD	Hardness 0.70 ± 0.21 GPa, elastic modulus 66.7 ± 2.4 GPa	Xiao et al. (2017)
TDP	DRNLGDSLHRQEI	%SMH_R_ of TDP increased; a significantly thicker and brighter remineralization layer with shallower lesions obtained	Ding et al. (2020)
P32	MPLPSYEVLTPLKWPSTDKTKREEVD	1.8-fold increase in elastic modulus and a 1.9-fold increase in hardness compared to demineralized enamel	Mukherjee et al. (2018)
QP5	QPYQPVQPHQPMQPQTKREEVD	%SMH_R_: 50.06, similar to NaF (58.48)	Wang Y. et al. (2020)
ADP5	SYENSHSQAINVDRT (AA sequence)	Vicker’s microhardness: 141 + 8 HV10; hardness: 2.23 + 0.23 GPa; elastic modulus: 58.6 + 4.7 GPa	Dogan et al. (2018)
Peptide-7	Asp-Asp-Asp-Glu-Glu-Lys-Cys	Ra and Rz: 19.0 ± 4.3 nm and 223.6 ± 23.6 nm; the hardness 497.79 ± 19.63; %SMH_R_: 84.13; adhesion force 63.80 ± 4.58 N	Liu et al. (2018)
Sp-H5	phosphoserine-DSHAKRHHGYKRKFHEKHHSHRGY	2.5-*μ*m-thick crystal layer is regenerated on the enamel surface	Zhou et al. (2020)
P-113-DPS	AKRHHGYKRKFH-SpSp	The thickness of the regenerated crystal layer in 24 h: 8.5 *μ*m	Zhou et al. (2021)
LCPS-CP	^37^SYSGYS^42^	Elastic modulus of 65.43 ± 15.57 GPa and surface hardness of 1.831 ± 0.5852 GPa for the LCPS-CP group	Chang et al. (2022)
8DSS	(Asp-Ser-Ser)_8_	lesions became shallower after pH cycling; shows remineralization results similar to 1 g/L NaF *in vitro* test	Yang et al. (2014)

Abbreviations: %SMH_R_, surface microhardness recovery ratio; ADP5, amelogenin derived peptide 5; LCPS-CP: LCPS-CP, low-complexity protein segments containing phosphonate group.

#### 3.3.1 Amelogenin analogs

Amelogenin analogs are created by mimicking the functional domain of amelogenin. These synthetic peptides outperform full-length amelogenin in synthesis, purification, and retention (Gungormus et al., 2012; Dogan et al., 2018). The focus of recreating enamel structure and function *in vitro* is inducing columns growth and elongation directly, which is predominantly regulated by the C-tail. Therefore, peptides with C-terminal can stimulate remineralization *in vitro*. Amelogenin inspired peptides of 26 and 32 amino acid residues (P26 and P32) with hydrophilic inner N- and C-terminal are produced to mimic the “nanosphere” structure in the enamel matrix (Mukherjee et al., 2018). A firm mineralized layer is successfully produced on the enamel surface after 7 days of *in-situ* culturing with polypeptide solution. *C*-axis oriented nanorods are generated on the enamel surface by repeating the peptide application process. P32 can restore the hardness of etched enamel better because the crystals created by P26 are smaller than those produced by P32. A chimeric peptide is created by grafting the C-terminal onto HA6-1, which can be selectively attached to the enamel surface (Xiao et al., 2017). The C-terminals of the chimeric peptide increases the peptide adsorption and facilitates the formation of a mechanically strong remineralized layer. QP5 is consisting of five highly conserved Gln-Pro-X repeat sequence in the center region and a hydrophilic C-tail (Lv et al., 2015; Chu et al., 2018; Ren et al., 2018; Li et al., 2020). When compared to amino acids, QP5 has a better remineralization impact, which effectively restored enamel surface hardness and reduced surface roughness value (Li et al., 2020; Wang Y. et al., 2020). Moreover, QP5 can enhance remineralization in a complicated oral environment, as demonstrated by the rat caries model (Han et al., 2017). Shortened amelogenin derived peptide 5 (shADP5) is employed as an active ingredient to generate a mineralized layer in solution. The enamel surface is healed after 1 h of mineralization, and the average hardness and elastic modulus are higher than control samples, with the hardness of 2.23 ± 0.23 GPa vs. 2.10 ± 0.26 GPa and elastic modulus of 58.6 ± 4.7 GPa vs. 55.1 ± 4.3 GPa (Dogan et al., 2018). A phase transfer lysozyme (PTL) membrane can be used to simulate the N-terminal of amelogenin (Wang D. et al., 2020). After the occurrence of amyloid aggregation, the internal structure of lysozyme is changed: the *α*-helixes unravel and the *β*-sheets is formed through hydrophobic interactions, which is similar to the spatial phase shift of amelogenin self-assembling. At the liquid/solid interface, those *β*-sheet-rich proteins are quickly organized into nanoparticles, forming a nanofilm that could be adsorbed on the enamel surface and serve as a scaffold for subsequent remineralization. The hydrophilic C-tail is then grafted onto PTL to produce PTL/C-AMG, which can guide HA growing in a direction. A 2.0–2.8 *μ*m thick remineralization layer is produced after applying 1 mg/ml PTL/C-AMG to demineralized tooth slices for 7 days. These remineralized layers have similar properties to natural enamel with a “fish-scale” structure.

#### 3.3.2 Statherin derived peptide

Statherin, a tyrosine-rich peptide with 43 amino acid residues, is a salivary protein that is found in the oral acquired membrane. Because of a unique combination of high negative charged domains on the N-terminal and enamel surface, statherin can securely cling to the enamel surface (Raj et al., 1992; Gururaja and Levine, 1996). To replicate the property of high HA binding, several peptides derived from statherin are created (Shuturminska et al., 2017; Luo et al., 2019; Carvalho et al., 2020). Separating the N-terminal of statherin can yield the peptide SN15 (Dodds et al., 2005; Shimotoyodome et al., 2006; Luo et al., 2019). Grafting SN15 onto PAMAM can improve its absorption on enamel surface (Gao et al., 2020). The statherin stimulated peptide and tannic acid are used to make SAP-TA (Yang et al., 2017). Polyphenol groups in TA can grab Ca^2+^ and trigger HA crystal renewal. Iron ions work in tandem with SAP-TA to generate a thick layer that boosts adsorption capacity. Therefore, SAP-TA/Fe (III) can improve the adhesion and mechanical properties of the remineralization interface (surface microhardness recovery >80%, binding force 64.85 N). Peptide-7 is designed and synthesized with a significant number of carboxyl groups on its side chain to help in firmly interacting with HA and directional elongation of HA crystals (Liu et al., 2018). Under the guidance of Peptide 7, a dense mineralized crystal layer with tight adhesion was formed.

#### 3.3.3 Antibacterial peptide inspired bioactive peptides

**FIGURE 6:**
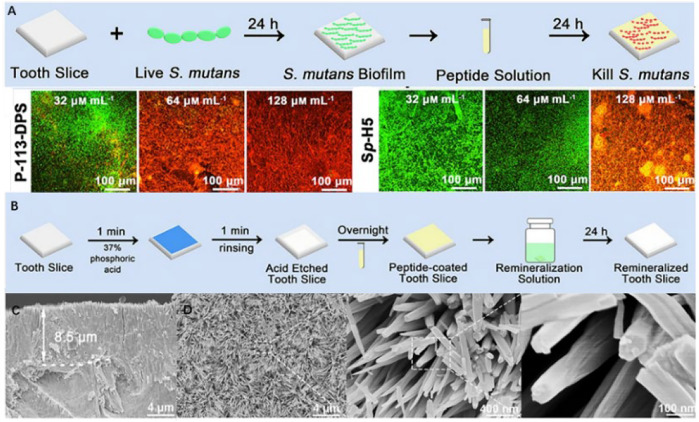
**(A)** Schematic diagram of anti-*S. mutans* biofilm on the enamel surface and fluorescence images (×20) of *S. mutans* biofilm in various concentrations of P-113-DPS and Sp-H5 solutions. **(B)** Remineralization experiment schematic. **(C)** 8.5 *μ*m needle-like remineralized layers formed by P-113- remineralized layers and **(D)** its FE-SEM micrographs (×5000, ×50,000, and ×200,000). Reprinted with permission from [ (Zhou et al., 2021) ]. Copyright ^©^ 2021 American Chemical Society.

**FIGURE 7:**
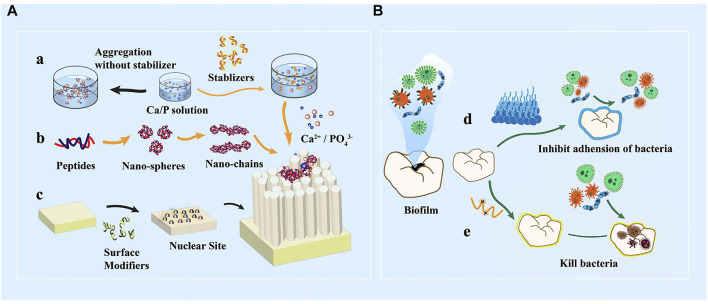
**(A)** *In vitro* peptide-induced bionic remineralization process. a) Organic matter stabilized calcium and phosphorus solution provides sufficient amount of ions b) Self-assembly of peptides into nanospheres, nano-chain structures orderly guide the deposition of calcium and phosphorus ions and extension of ordered orientation c) Organic matter modifies the substrate surface to form nucleation sites. **(B)** Mechanism of action of antibacterial anti-caries materials. d) Enamel surface coating reduces microbial adhesion; e) Positively charged peptides direct killing of caries-causing bacteria.

Bioactive peptides have antibacterial and remineralization properties, which can be obtained by grafting units capable of promoting remineralization onto the active sequences of antimicrobial peptides. This bioactive peptide can protect enamel against demineralization while also promoting self-healing regeneration in a remineralized environment. P-113 is the smallest antibacterial unit of histatin 5, which is a type of natural antimicrobial peptide (Zhou et al., 2020). A study coupled dopamine (DA), SpSp (DPS) domains, and binding peptide binding peptide SKHKGGKHKGGKHKG on P-113 to find the most cost-effective peptide (Zhou et al., 2021). The experiment has discovered that P-113-DPS show similar antibacterial effect to Sp-H5 and can kill the majority of *Streptococcus mutans (S. mutans)* at low concentrations. After a 24-hour remineralization experiment, an 8.5 *μ*m thick needle-like remineralization layer is formed on the enamel surface in the P -113-DPS group, twice as thick as the control group (4 *μ*m) (Figure 6). The low-complexity protein segments (LCPSs) ^37^SYSGYS^42^ in the fused in sarcoma protein is capable of forming nucleation structures that form reversible amyloid fibrils (Hughes et al., 2018). LCPSs are highly hydrophilic and structurally flexible. Due to weak multivalent interactions, proteins are entangled and subsequently form web-like structures. LCPSs containing a phosphate or phosphonate group is named LCPS-OP and LCPS-CP. These acidified polypeptides can bind calcium ions and acts as soft templates to induce HA formation. At the same time, the hydrophilic negatively charged peptide coating can reduce the bacterial adhesion of caries-causing bacteria by virtue of the negative electric mutual repulsion (Chang et al., 2022). Given that this bioactive peptide may more effectively repair damaged enamel while inhibiting further erosion of dental cariogenic bacteria, it may be an ideal material for the prevention of dental caries. In addition, some antimicrobial materials have been added to the remineralization system to promote enamel remineralization. The first type of materials can cover the enamel surface with an antifouling layer, and the second type can be used to destroy the bacterial biomass through the positive charges. Table 2 summarizes these materials that combine antimicrobial and remineralization functions and Figure 7 shows their modes.

**TABLE 2:** Antibacterial remineralization dual materials.

Materials	Antibacterial mechanism	Bacterial used	Antibacterial adhesion results	Antibacterial test results	References
PASP-PEG	The PEG film on the enamel surface is resistant to bacterial adhesion	*S. sanguis/S. mutans*	Almost no bacterial attachment is detected on the surface	NA	Hou et al. (2020)
ZHA@ALN-PAA	The released Zn^2+^ are antibacterial	*S. mutans UA159*	NA	IR: 75.05%	Xu et al. (2020)
Sp-H5	The cationic amino acid residues in H5 bind to cell wall, enhance membrane permeability and interact with intracellular DNA of *S. mutans* to induce cell death	*S. mutans*	Viability counts at 16× MIC [6.11E+05 (CFU/ml)]	MIC: 2 μmol/ml	Zhou et al. (2020)
				MBC: 4 μmol/ml	
P-113-DPS	P-113-DPS crosslink with bacterial membrane phospholipids, increasing membrane permeability and forming perforation, preferentially occupy the binding site to inhibit the adhesion of *S. mutans*	*S. mutans*	Viable counts of *S. mutans* in P-113-DPS-coated [2.03E+05 (CFU ml^−1^)]	MIC:8 μM ml^−1^	Zhou et al. (2021)
				MBC:16 μM ml^−1^	
LCPS-CP	Hydrophilic LCPSs eliminate adsorbed biomolecules by forming an anti-sewage ensemble; negatively charged phosphate coatings cause electrostatic repulsion between the bacterial film and the enamel, ultimately reducing adhesion	*S. mutans*	relative biomass value of the no peptide and LCPS-OH are more than eightfold greater of LCPS-OP and LCPS-CP	NA	Chang et al. (2022)
CMC/ACP	CMC neutralizes the negative charge on the surface of bacteria through a large number of positive charges to reduce the early adhesion of bacteria	*S. mutans/S. Gordonii*	adherence of *S. mutans* inhibited by 89.7%; *S. gordonii* by 86.1%	Biofilm formation decreased by 45.3% (*S. mutans*) and 44.0% (*S. Gordonii*)	He et al. (2019)
CS-QP5	CS captures free hydrogen ions, slows pH fall, damages the bacterial cell wall, and causes bacterial death	*S. mutans*	inhibited adhesion up to 95.43%	MIC/MBC: 5 mg/ml ^−1^	Ren et al. (2019)
PAMAM-NH_2_	PAMAM-NH_2_ destroy the bacterial wall by contacting bacteria for sterilization, improve the smoothness of remineralized layer and reduce bacterial adhesion	*S. mutans UA159*	Bacterial adhesion forces 3.64 ± 1.52 nN (control group: 5.52 ± 1.6 nN)	Colony-forming unit counting 5.78 ± 0.27 (control group: 6.13 ± 0.2)	Jia et al. (2020)

Abbreviations: NA, not available; PASP-PEG, poly (aspartic acid)-polyethylene glycol; ZHA@ALN-PAA, zinc-substituted hydroxyapatite/alendronate-grafted polyacrylic acid; IR, inhibition ratio; MIC, minimal inhibitory concentration; LCPS-CP, low-complexity protein segments containing phosphonate group; MBC, minimal bactericidal concentration.

#### 3.3.4 Dentin phosphoprotein derived peptide

Dentin phosphoprotein, made up of a significant amount of aspartate serine repeat sequences that have a strong affinity for HA, can act as a nucleation template in the process of dentin mineralization (He and George, 2004). Therefore, inspired by dentin phosphoprotein, the peptide 8DSS containing eight DSS repetitions is synthesized. 8DSS can capture Ca^2+^ and act as a diffusion barrier that prevent CaP from dissolving. In the repair of both surface cavities and deep lesions, 8DSS demonstrated equal remineralization ability to NaF (Yang et al., 2014; Liang et al., 2015; Zheng et al., 2019). In addition, 8DSS is able to resist hydrolase assault and sustain its action in the mouth due to the short peptide chain length, which is conductive to clinical application.

#### 3.3.5 Self-assembly peptide 11-4 (P_11_-4)

Self-assembly peptide P_11_-4 is a well-studied small molecule peptide. When activated by external stimuli, P_11_-4 can self-assemble through intermolecular hydrogen bonds between peptide backbones and form three-dimensional scaffolds in lesions (Alkilzy et al., 2018a). At this time, the negative group formed by 4 Glu-residues on P_11_-4 can attract calcium ions and induce mineralization. According to µCT imaging, the mineralization of the samples treated by P_11_-4 increase by 68% in 14 days (Kind et al., 2017). It is worth noting that P_11_-4 guided remineralization occurs in the subsurface of lesions, which can compensate for the shortcomings of fluoride. As a result, the combination of P_11_-4 and fluoride varnish can produce good results in clinical applications. In an experiment on children over 5 years old with obvious active early caries, P_11_-4 + fluoride varnish are superior to fluoride in terms of vision and safety (Alkilzy et al., 2018b). In some other clinical trials, P_11_-4 successfully treated white spot lesions caused by enamel demineralization and significantly improved the appearance (Doberdoli et al., 2020; Jablonski-Momeni et al., 2020; Sedlakova Kondelova et al., 2020).

### 3.4 Alendronate

Alendronate (ALN), a powerful bone resorption inhibitor with a high affinity for HA, is used to treat and prevent osteoporosis (Chen S. et al., 2020). The phosphate of ALN exchanges with the phosphate of HA in enamel, forming coordination chains that tightly bind it to the enamel surface (Palazzo et al., 2007). Therefore, ALN can act as a “glue” in the mineralization system to increase the adsorption of materials. ALN modified poly (amino amine) dendrimers (Wu et al., 2013) and carboxymethyl chitosan (Wang et al., 2017) can significantly increase their absorption on the enamel surface. In addition, after forming a HA layer around the ALN-modified polyacrylic acid (PAA), the outer layer of HA is zinc-modified to synthesize ZHA@ALN (Xu et al., 2020). Once ZHA@ALN dissolved by acid, a substantial number of calcium, phosphorus, and zinc ions can be released for remineralization and sterilizing. The inner layer of ALN-PAA adheres quickly to the enamel surface due to the ALN. Then, PAA serves as an antifouling layer with 75.05% bacteriostatic efficiency.

## 4 Polymer materials

Polymer materials have complex side groups and spatial structures, which enable them to mimic the enamel matrix and induce mineralization. Some are used to keep ions stable, and some can be made into gel carriers to transport functional peptides while forming a protective layer on the enamel surface. Due to their high biocompatibility and adaptability, polymer materials can be an ideal choice for promoting enamel remineralization.

### 4.1 Non-collagenous protein analogs

Non-collagenous proteins (NCPs) stabilize crystal precursors during dentin and bone collagen mineralization. NCP analogs, such as polyaspartic acid (pAsp), polyglutamic acid (PGA), and biocompatible polymers polyacrylic acid (PAA), contain a large number of carboxyl groups. These polymers can be used to create induced liquid precursors by stabilizing calcium ions. PGA and pAsp can also bind to calcium ions on the enamel surface, strengthening adhesion and providing nucleation sites (Ustriyana et al., 2020a; Ustriyana et al., 2020b). It has been discovered that the *α*-helical of pAsp or PGA can promote HA crystal nucleation by templating Ca^2+^ distribution. The HPO and -COO- can work together to attract Ca^2+^ and form stable Ca^2+^ triangles, which can develop into the nucleation core of ACP (Zeng et al., 2021). PAA can also chelate with Ca^2+^ while maintaining liquid phase stability and transporting ions continuously for subsequent biomineralization (Chen R. et al., 2020; Xu et al., 2020; Li N. et al., 2021). Furthermore, PAA can direct the transformation of ACP to form acicular microcrystals (Wang et al., 2018).

### 4.2 Poly (amino amine) (PAMAM)

Poly (amino amine) (PAMAM) is a kind of synthetic protein with a dendritic structure. PAMAM can self-assemble into nanospheres, nanochains, and nanoribbons (Yang et al., 2015). Grafting different groups such as -NH_2_, -COOH and -OH onto PAMAM can improve its ability to bind Ca^2+^or promote its adsorption on the enamel surface. The mineralization effects decrease in the order of -NH_2_ > -COOH > -OH (Fan et al., 2020). This is because positive charged PAMAM-NH_2_ can be more adsorbed on the negatively charged enamel surface. In addition, the adherence of *S. mutans* is also evaluated. Both PAMAM-COOH and PAMAM-NH_2_ are shown to be effective in forming a smooth remineralized layer and minimizing *S. mutans* adherence (Jia et al., 2020). Grafted with SN15, SN15-PAMAM can increase adsorption on the enamel surface and achieve 90% higher mineralization effect than the control group. (Gao et al., 2020).

### 4.3 Polydopamine

Polydopamine (PDA), the polymer of dopamine that rich in amino and phenolic groups, shows great hydrophilicity. It has been used as a functional agent to increase the wettability and biocompatibility of substrate (Barclay et al., 2017; Ghorbani et al., 2019). After being immersed in PDA solution, a dense film can be formed on the surface of the material in a short time (Kaushik et al., 2020). The film contains a large number of charged groups, to which calcium and phosphorus ions will be attracted and form a stable bond (Ryu et al., 2010; Murari et al., 2020). It is also observed that the HA crystals on the PDA modified enamel surface accumulated more closely, suggesting that PDA might help in inducing uniform crystal nucleation (Zhou et al., 2012). This may be because PDA can increase surface hydrophilicity, decrease the interfacial energy, and accelerate crystallization speed of HA (Qu et al., 2020). Based on the super adhesion of PDA, HA layer can be synthesized on the subsurface of different materials after being modified (Chen et al., 2019; Wong et al., 2022).

### 4.4 Biopolymers

Biopolymers, including proteins and polysaccharides, have been used for the bionic formation of HA. Most of these polymers are mostly used in mineralized systems in the form of gels. All of these biopolymers show excellent non-immune and biocompatible properties, meanwhile with the advantages of easy storage and clinic application.

Chitosan (CS), a cationic polysaccharide, can rarely produce allergic or inflammatory reactions in humans (Younes and Rinaudo, 2015). Therefore, CS has been used to construct organic templates and scaffolds, which can ensure the bioactivity of peptides for mineralization guidance (Ruan et al., 2016; Ren et al., 2018). Furthermore, CS is also able to prevent bacteria from adhering and reproducing. The adherence of *S. mutans* may be decreased by 94.91% by employing CS alone (Ren et al., 2019). This is because chitosan is positively charged. When CS comes into touch with the negatively charged bacterial wall, the structure of the bacterial wall would be disrupted. Simultaneously, positive charged CS can adhere to the negatively charged enamel surface, preventing acid erosion (He et al., 2019; Boda et al., 2020). In addition, the antibacterial function can be enhanced when CS paired with fluoride (Wassel and Khattab, 2017; Ren et al., 2019). Carboxymethyl chitosan (CMC), formed by CS carboxylation, also has excellent ACP stabilization and can promote the formation of enamel remineralization layers. (Chen et al., 2015; Xiao et al., 2017). The mineralization system using chitosan as a gel carrier is summarized in Table 3.

**TABLE 3:** Summary of chitosan basing remineralization systems.

Materials	Approach	Remineralization effect	Reference
CS-AMEL	15 min CS-AMEL 2 times per day; 8 h modified remineralization solution, 16 h remineralization solution	The depth of caries decreased from ∼100 to ∼30 μm	Ruan et al. (2016)
CMC-ALN/ACP + GLY	10 min CMC-ALN/ACP + Gly, remineralization solution per day for 7 d	%SMH_R_: 49.4; Modulus recovered by 68.6%	Wang et al. (2017)
MMP-20–CS-AMEL	MMP-20–CS-AMEL hydrogel 15 min; AS with 1 ppm at 37°C, 5 d	Obtained a 2.4-fold increase in hardness and 2.6-fold increase in modulus	Prajapati et al. (2018)
CS-QP5	2.5 mg/ml CS-QP5 for 5 min, 4 times daily; remineralization and demineralization solution alternated for 12 d at 37°C, low-speed magnetic stirring (100 rpm)	%SMH_R_: 50.6; Modulus recovered by 68.6%	Ren et al. (2019)
CMC/LYZ-ACP	CMC/LYZ-ACP nanogels 10 min; 0.15 M 30 s; Tris−HCl buffer at pH 8 for 30 min	The hardness is 3.8 ± 0.3 Gpa, Modulus 80.3 ± 5 GP; compared to the nature enamel group hardness of 4.3 ± 0.5 Gpa; modulus 89.5 ± 5.1 Gpa	Song et al. (2021)
CS-A hydrogels	1 M CaCl_2_ 15 min, CS-A hydrogel 2 h, AS at 37°C, 7 d	A layer of 7.5–8.5 µm thick for 7d; the hardness 2.26 Gpa, and %SMH_R_ reached 77.4	Musat et al. (2021)

Abbreviations: %SMH_R_, surface microhardness recovery ratio; CS-AMEL, chitosan-amelogenin; CMC-ALN/ACP + Gly, carboxymethyl chitosan-alendronate/amorphous calcium phosphate; MMP-20–CS-AMEL, matrix metalloproteinase-20- chitosan-amelogenin; CMC/LYZ-ACP, carboxymethyl chitosan/lysozyme-amorphous calcium phosphate; CS-A hydrogels, chitosan (CS) and agarose (A) hydrogels.

Agarose is a natural polysaccharide with -OH groups that can form a thermally reversible gel. Agarose aqueous gel is widely used in medical systems such as mineral regeneration and drug delivery (Zarrintaj et al., 2018). The abundant hydroxyl groups in agarose molecules have a strong mutual attraction with Ca^2+^, allowing agarose to control the formation of nano ACP precursors and act as an ion reservoir to transport mineral precursors to the enamel surface for mineral mesomorphic transformation. The average elastic modulus and nano hardness of enamel increased significantly to 89.46 ± 11.82 and 3.04 ± 0.75 GPA after 6 days of the interaction of agarose gel with 500 ppm F (Cao et al., 2014a). When chitosan is added to the agarose aqueous gel, the groups between the two gels are cross-linked with each other, forming a fiber structure together with calcium ions, which can further simulate the protein matrix for enamel repair. The regeneration layer is 7.5–8.5 *μ*m thick and regained 77.4% of the natural enamel’s microhardness (Musat et al., 2021). Agarose can also be combined with amelogenin to form oriented hexagonal prism enamel columns on the enamel surface (Cao et al., 2014b).

Gelatin is a peptide molecular polymer. Gel peptides in gelatin can form salt bonds with phosphate groups on the surface of apatite, causing enamel-like minerals to regenerate. The limited directional diffusion of ions in the gel environment promotes heterogeneous nucleation, resulting in a crystal with a clear structure (Zhang et al., 2010). Using the bionic double-layer gel system assisted by anodic aluminum oxide, it is possible to successfully prepare HA crystals with good orientation (Chen et al., 2019).

Silk fibroin (SF) governs the synthesis of calcium carbonate in mollusks and the creation of animal shells. SF contains a large number of *β*-sheets, which are rich in acid aspartic acid and have a high affinity for calcium ions. In the rotary thermal evaporation approach, SF serves as a template to guide heterogeneous nucleation of HA and mineral layers with natural enamel-like shape, organization, and mechanical characteristics swiftly formed (Wang S. et al., 2020).

Abalone water-soluble matrix (AWSM) plays an important role in the formation abalone shells. The proportion of organic matter and inorganic minerals in abalone shells is very similar to enamel (95% calcium carbonate and less than 5% organic matter). Therefore, AWSM can promote the formation of crystals. High AWSM concentration can increase the content of calcium and phosphate in the mineralized layer and promote to form a parallel, dense and highly ordered structure (Wen et al., 2016).

### 4.5 Other polymers

Carboxy betaine (CB) polymers are amphoteric polymers with functional carboxyl and quaternary ammonium groups. The carboxyl groups can serve as Ca^2+^ and PO_4_^3−^ deposition sites, whereas the positive quaternary amino group has bactericidal properties (Xu et al., 2018). Furthermore, CB can resist bacterial adhesion via electrostatically induced hydration. ACP can be stabilized by amphoteric ionic poly (carboxy betaine acrylamide) (PCBAA). PCBAA/ACP nanocomposites contribute to the growth of HAP in the damaged sublayer of enamel by blocking spontaneous ion conversion on the enamel surface while releasing sufficient ions (He et al., 2022). Thus, PCBAA/ACP nanocomposites performed admirably in both remineralization (10.08 *μ*m thick remineralized layer in mice intraoral for 14 days) and antimicrobial experiments (almost no bacterial adherence). ACP and poly (vinylpyrrolidone) nanofibers are mixed, and making electrospun mats. The mats can be hydrated to form a gel in the savila containing fluoride. Then, calcium and phosphorus ions crystallize under the guidance of fluorine ions to form HA (Fletcher et al., 2011).

## 5 Conclusion and perspective

Enamel caries have been common problem in our daily life, great efforts have been paid to design new materials and realize the remineralization of enamel. However, it is still a great challenge to repair the defect enamel and restore its functions, as for the emerging materials for enamel remineralization, there is still a long way to go to satisfy the clinic applications. Firstly, most of the current materials used for the remineralization still need a long time, from several days to more than 10 days, secondly, the stability and mechanical properties are not satisfying enough, in addition, most of the remineralization systems depends a lot on the solution or concentration of mineralization medium. Therefore, it is critical important to design advanced materials that can be used in enamel remineralization and solve the clinic problems.

In the next decades, materials, both inorganic materials or polymers that can promote the mineralization speed, especially which could tune the alignment of mineralized apatite along the native mineral structure or composition, should be a charming field, besides, on considering the complicated oral environment, bacterial infections also threaten the treatment of dental health, therefore, materials with multifunction should also be designed and may pave the way of enamel remineralization. In addition, further researches in the remineralization mechanisms are also much important, which may be helpful to direct the design of new materials and their final applications.
